# Annual Research Review: The nature and classification of reading disorders – a commentary on proposals for DSM-5

**DOI:** 10.1111/j.1469-7610.2011.02495.x

**Published:** 2011-12-05

**Authors:** Margaret J Snowling, Charles Hulme

**Affiliations:** 1 Department of Psychology, University of YorkYork, UK; 2 Division of Psychology and Language Sciences, University CollegeLondon, UK

**Keywords:** Reading disorders, language disorders, dyslexia, reading comprehension impairment, intervention

## Abstract

This article reviews our understanding of reading disorders in children and relates it to current proposals for their classification in DSM-5. There are two different, commonly occurring, forms of reading disorder in children which arise from different underlying language difficulties. Dyslexia (as defined in DSM-5), or decoding difficulty, refers to children who have difficulty in mastering the relationships between the spelling patterns of words and their pronunciations. These children typically read aloud inaccurately and slowly, and experience additional problems with spelling. Dyslexia appears to arise principally from a weakness in phonological (speech sound) skills, and there is good evidence that it can be ameliorated by systematic phonic teaching combined with phonological awareness training. The other major form of reading difficulty is reading comprehension impairment. These children read aloud accurately and fluently, but have difficulty understanding what they have read. Reading comprehension impairment appears to arise from weaknesses in a range of oral language skills including poor vocabulary knowledge, weak grammatical skills and difficulties in oral language comprehension. We suggest that the omission of reading comprehension impairment from DSM-5 is a serious one that should be remedied. Both dyslexia and reading comprehension impairment are dimensional in nature, and show strong continuities with other disorders of language. We argue that recognizing the continuities between reading and language disorders has important implications for assessment and treatment, and we note that the high rates of comorbidity between reading disorders and other seemingly disparate disorders (including ADHD and motor disorders) raises important challenges for understanding these disorders.

## Introduction

Becoming literate opens doors to education, employment and perhaps ultimately adult well-being. All children in literate societies have a right to be literate, and in developed educational systems, the expectation is that by the end of primary schooling a child can read fluently with understanding, so that they can ‘read to learn’ (Education for All, Fast Track Initiative, 2011). Indeed, there is evidence that, at least in the English language, reading skills reach an asymptote at around this age (Francis, Shaywitz, Stuebing, Shaywitz, & Fletcher, 1996), and further development depends primarily upon increased exposure to print through practice. Against this backdrop, there are longstanding concerns about children who have reading (and writing) difficulties in our school systems. Thus, it might be argued that reading difficulties are the domain of education and do not warrant a place in psychiatric manuals. However, disorders of reading are commonly associated with other neurodevelopmental disorders including disruptive behavioural disorders (Maughan, Pickles, Hagell, Rutter, & Yule, 1996), and levels of anxiety are frequently raised in people with dyslexia (Carroll & Iles, 2006). It follows that, mental health professionals need to be aware of the nature and developmental course of reading disorders, so that they may consider their potential contribution to child and adult well-being.

Broadly, there are two forms of reading disorder: difficulties with decoding (dyslexia) and difficulties with comprehension (Cain, 2010; Hulme & Snowling, 2009). These different reading disorders have different causes and require different treatments. In this article, we discuss the proposed classification of reading disorders in DSM-5 and highlight a concern that the focus is on dyslexia. We agree with Pine et al. (in press) that it is important for DSM-5 to take a developmental perspective, and from this standpoint, it is necessary to recognize the continuities between language and reading disorders as well as co-morbidities with other disorders that may increase the risk of learning disorder. We also stress that neither disorders of reading accuracy (dyslexia) nor of reading comprehension (‘reading comprehension impairment’) lend themselves to categorical diagnosis. Rather, an understanding of multiple risk factors and causal mechanisms is essential if practitioners are to ensure that children’s reading difficulties are identified early, and timely interventions are put in place.

## DSM-5 proposals for the classification of reading disorders

Researchers, clinicians and educators would agree that, among learning disorders, reading disorders have a significant impact on educational achievement throughout life. In DSM-5, the term ‘reading disorder’ is not used, but the category ‘Neurodevelopmental Disorders’ lists Learning Disorders and Communication Disorders amongst a variety of other disorders that have their onset during the preschool or early school years. There are three forms of Learning Disorder and seven forms of Communication Impairment, but what is not clear from the listing in DSM-5 is that there are inter-relationships between the different disorders.

Learning Disorder is the generic term used in DSM-5 to describe disorders that are characterized by difficulties in learning academic skills and which significantly affect academic achievement or daily functioning if accommodations are not made. The Learning Disorders are Dyslexia, Dyscalculia and Disorders of Written Expression. The Communication Disorders include Language Impairment, Specific Language Impairment and Speech-Sound Disorder, all of which have been associated with dyslexia, suggesting either they are developmental variants of the same disorder or the behavioural outcomes of shared risk factors. In DSM-5, the relationships between reading, speech and language impairments are not transparent, even though how best to conceptualize this has been the subject of much recent research (Bishop & Snowling, 2004; Pennington & Bishop, 2009 for reviews).

Dyslexia is an impairment that affects the development of decoding skills in reading, and in DSM-5 this term replaces ‘reading disorder’. Here, ‘decoding’ refers to the component reading skills that reflect the ability to map phonology (the speech sounds of words) onto orthography. Decoding depends upon letter knowledge and phonological skills primarily, and is usually assessed by measures of word identification (reading aloud words) and phonological decoding skills (reading aloud nonwords – a test of a child’s ability to generalize knowledge to items that have not been directly taught). Children with dyslexia typically have difficulties in learning to read accurately and with adequate speed (fluency). Moreover, even when adequate word reading accuracy is achieved, fluency deficits are much less easy to remediate (Fletcher, Lyon, Fuchs, & Barnes, 2007). The proposed definition of dyslexia for DSM-5 (DSM-5, 2010) broadly reflects this characterization:

1. Difficulties in accuracy or fluency of reading that are not consistent with the person’s chronological age, educational opportunities or intellectual abilities.
2. The disturbance in criterion 1, without accommodations, significantly interferes with academic achievement or activities of daily living that require these reading skills.

It is notable, however, that spelling problems are not mentioned, and yet people with dyslexia typically have spelling (and writing) difficulties that are often more severe and more persistent than their problems with reading (Bruck, 1990; Maughan et al., 2009).

In contrast to dyslexia that affects word-level decoding skills, ‘poor comprehenders’ decode well, but have difficulties in understanding what they read. As a result, there is a marked discrepancy between scores on standardized tests of reading accuracy (which are in the normal range) and reading comprehension. The evidence we have indicates that difficulties with reading comprehension are relatively common, and as many as 10% of unselected samples of children satisfy the criteria for this ‘diagnosis’ (Hulme & Snowling, 2011).

Reading comprehension impairment was not separated from decoding difficulty in the *Diagnostic and Statistical Manual of Mental Disorders*, 4th Edition (American Psychiatric Association, 1994); both dyslexia and reading comprehension impairment would be classed as reading disorders because they reflect ‘educational under- achievement..[in] reading accuracy *or* comprehension’. A positive change proposed for DSM-5 has been the decoupling of impairments of decoding from impairments of comprehension. However, it is of concern that the current draft recommends that reading comprehension impairment per se be omitted, and rather than counting it as a distinct form of reading disorder, it is considered as a form of Language Impairment. Contrary to this, we argue that it is important to take account of a large body of evidence showing that different forms of reading disorder are associated with different ‘cognitive phenotypes’. Understanding these is important in order to understand the risk factors that lead to reading disorders and to motivate appropriate interventions.

We begin by considering the process of learning to read to provide a framework for considering what places a child at risk of reading failure. We proceed by reviewing evidence showing that many children whose primary diagnosis is a Communication Disorder also experience a variety of forms of reading difficulty. Thus, reading and language impairments are strongly inter-connected and this should be reflected in DSM-5. More generally, we shall show that a ‘categorical’ approach to Learning Disorders has limitations, and from an educational perspective, considering the dimensions which underpin reading and language, and how they interact, has more direct implications for assessment and teaching.

## A developmental framework for considering disorders of reading

### Learning to decode

Reading is a complex skill that depends upon a range of cognitive and linguistic abilities. Fundamentally, the process of learning to read requires the development of a system of mappings between the visual symbols of the writing system and the pronunciations of words. However, the ease with which children learn to read depends upon the language in which they are learning (Ziegler & Goswami, 2005). Although research on reading has been dominated by the study of the English language (Share, 2008), there is now considerable evidence that the regularity or ‘transparency’ of a writing system (orthography) – how consistently the letters or characters of the language map to speech sounds (phonology) – determines how easily children learn to read. Cross-linguistic comparisons have shown that learning to read (to decode print) is faster in transparent alphabetic orthographies like German, Italian and Finnish than in English (Seymour, Aro, & Erskine, 2003), but the predictors of reading skill are the same (Caravolas, Volín, and Hulme, 2005; Ziegler et al., 2010). Less is known about learning to read in alphasyllabaries, such as Korean and many of the southern Indian languages (Nag, Caravolas, & Snowling, 2011), or in logographic languages, such as Chinese (Shu, Chen, Anderson, Wu, & Xuan, 2003) and Japanese kanji (Uno, Wydell, Haruhara, Kaneko, & Shinya, 2009). These orthographies have extensive symbol sets that pose a considerable challenge, making learning to read a protracted process.

With findings such as these as a backdrop, the consensus view for many years has been that dyslexia has its origins in a phonological deficit (Vellutino, Fletcher, Snowling, & Scanlon, 2004). The phonological deficit (or endophenotype) has a direct causal influence on learning to read, because in an alphabetic orthography, the ability to map between letters (graphemes) and phonemes is compromised (Hulme & Snowling, 2009; Chapter 2). There is a growing body of evidence that this deficit may be universal – hence, in logographic languages like Chinese, dyslexia is also associated with phonological deficits (Ho, Chan, Lee, Tsang, & Luan, 2004; McBride-Chang et al., 2008). Moreover, phonological deficits are observed in dyslexia across the life span, even in individuals who have overcome their difficulties and read adequately (Hatcher, Snowling, & Griffiths, 2002; Ramus et al., 2003).

The phonological deficit hypothesis accounts for a wide range of behavioural symptoms associated with dyslexia. Verbal short-term memory impairments that reflect phonological coding deficits are also seen in dyslexia alongside problems of phonological learning that may affect the learning of new words and foreign languages (Snowling, 2000). The findings of behavioural studies that support the phonological deficit hypothesis concur with evidence from genetic and neuroimaging studies; these highlight that reading and phonological skills share genetic variance (Pennington & Olson, 2005 for a review), and that the brain systems that underpin reading also subserve phonological processing (McCrory, Mechelli, Frith, & Price, 2005). A related deficit, currently the subject of intensive research, is rapid naming, the ability to name familiar items (objects, colours, letters and digits) rapidly. Although this task taps several cognitive skills (e.g. retrieval of phonological codes, speed of processing and executive function), a plausible hypothesis is that the same brain systems involved in mapping between visual and phonological codes for picture naming also underlie the process of mapping between printed words and their pronunciations (Blau, van Atteveldt, Ekkebus, Goebel, & Blomert, 2009; Lervåg & Hulme, 2009).

The phonological deficit hypothesis is reflected in two contemporary definitions of dyslexia which have guided educational policy, but it is not mentioned in DSM-5. The definition of the US National Institutes of Child Health (National Institute of Child Health, 2002) states: ‘Dyslexia.... is characterized by difficulties with accurate and/or fluent word recognition, and by poor spelling and decoding abilities. These difficulties typically result from a deficit in the phonological component of language that is often unexpected in relation to other cognitive abilities and the provision of effective classroom instruction’. Along similar lines, the Rose Review that examined the identification of and support for literacy problems in England (Rose, 2009) proposed a working definition: ‘Dyslexia is a learning difficulty that primarily affects the skills involved in accurate and fluent word reading and spelling. Characteristic features of dyslexia are difficulties in phonological awareness, verbal memory and verbal processing speed’.

Acknowledging phonological deficits within the definition of dyslexia is important for several reasons. First, as phonological development is an aspect of spoken language skill, it highlights the co-morbidity between Communication Impairments and word-level (decoding) reading difficulties. Second, the phonological ‘endophenotype’ is present before reading instruction and hence can be considered as a marker of risk or liability; third, the impairment provides a rationale for an intervention that includes training in phonological skills.

### The development of reading comprehension

The first step to reading comprehension is decoding. Beyond decoding, reading comprehension requires access to the meanings of words and higher level processes such as sentence integration, inferencing and comprehension monitoring (the reader’s ability to detect when comprehension of a passage has broken down); all of these skills need to be brought to bear to develop a mental representation of the text (Kintsch, 1998; Kintsch & Rawson, 2005; for reviews). Although it can be assumed that many of these processes are in place in the developing child – who for many years has been listening and understanding spoken language – the child must hone these skills and use them in concert to read fluently with sufficient proficiency to ‘read to learn’ (Perfetti, Landi, & Oakhill, 2005).

A model that has been influential as a framework for thinking about the distinction between difficulties that affect decoding and those which affect reading comprehension is the Simple View of Reading (Gough & Tunmer, 1986). According to the Simple View, Reading Comprehension is the product of Decoding Ability and Linguistic Comprehension. Thus, adequate reading comprehension depends critically upon both the ability to decode print (translate written language into speech) and to understand spoken language. There is good evidence from studies of typically developing children that variations in reading comprehension skills are strongly predicted by variations in decoding and listening comprehension. In addition, behaviour genetic evidence suggests that both word recognition and listening comprehension are subject to genetic influence, and that these genetic influences, in turn fully account for individual differences in reading comprehension (Keenan, Betjemann, Wadsworth, DeFries, & Olson, 2006). Finally, as children get older, the correlation between reading comprehension and decoding skills tends to decrease, whereas the correlation between reading comprehension and listening comprehension increases – suggesting that at older ages, reading comprehension comes to depend relatively more on language comprehension ability and less on the ability to decode print which is less more of a ‘bottleneck’ for younger children (Gough, Hoover, & Peterson, 1996).

A great deal of evidence suggests that ‘poor comprehenders’ have weak oral language comprehension. Two key forms of linguistic knowledge that underlie comprehension are semantics (the system of language concerned with word meanings) and grammar (the system of language concerned with how words and word segments (morphemes) are combined to convey meaning). Several studies indicate that children with reading comprehension impairment display broad language difficulties that include weak vocabulary knowledge, difficulties in processing grammatical information in spoken language and poor performance on general measures of language comprehension (Catts, Adlof, & Ellis Weismer, 2006; see Nation, 2005 and Hulme & Snowling, 2011 for reviews). However, they have better developed phonological skills. For most of these children, their language difficulties are not severe enough for them to be diagnosed as having language impairment, but a reasonable view would be that most have a sub-clinical language difficulty that is manifested clearly in their reading comprehension problems.

In addition, to underlying language weaknesses, a wide range of other explanations for these children’s reading comprehension difficulties have been considered including deficits of working memory, problems in making inferences, problems in comprehension monitoring and pragmatic language deficits (Cain, 2010; Hulme & Snowling, 2009; Ricketts, 2011). The extent to which these originate in processes that are separable from more basic problems with language is debated. Nonetheless, it is clear that reading comprehension demands a high degree of executive control not least to focus on the main themes of a text and to suppress irrelevant information (Gernsbacher & Faust, 1991). To the extent to which difficulties with executive processes are observed in conditions such as ADHD and autism spectrum disorders (ASD), they may contribute to the problems of reading comprehension that are sometimes observed in these disorders (McInnes, Humphries, Hogg–Johnson, and Tannock (2003); Henderson, Clarke, & Snowling, 2011; Ricketts, 2011). The fact that reading comprehension impairment is a feature of other disorders might be taken to imply that it is not a distinct disorder; however, recognizing that it can be the outcome of a number of different developmental trajectories associated with different risk factors does not lessen its impact on academic achievement, and therefore, we would argue that it should be appropriately classified as a Learning Disorder.

### The development of spelling

Learning to spell has attracted much less research attention than learning to read, although in general, it is the more difficult process. The early development of spelling draws on remarkably similar processes to reading, namely letter-sound knowledge and phoneme awareness (Caravolas, Hulme, & Snowling, 2001; Muter, Hulme, Snowling, & Taylor, 1998). However, it is important to remember that the two skills are not identical; the consistency of grapheme–phoneme relationships is only partly mirrored in phoneme–grapheme mappings, and in most languages, learning to spell demands greater knowledge of orthographic and morphological conventions than does learning to read (Treiman & Kessler, 2005).

Orthographic conventions are embodied in the abstract knowledge that readers possess about the writing system, such as the fact that, in English, a word cannot start with ‘ck’. Children become aware of the orthographic consistencies that characterize their language either through reading or via direct instruction with feedback from a teacher (Nunes & Bryant, 2009). According to Caravolas et al. (2001), it is only during the second year of reading instruction that reading begins to influence spelling, and thereafter, it is reasonable to suppose that increasing reading experience brings with it sensitivity to morphological structure (the meaning components of words). Morphological boundaries can also give information about inconsistencies in how phonology maps to orthography (Treiman & Cassar, 1997); for example, the past tense inflection is pronounced/t/, but written ‘ed’, and the affix ‘cian’ is used at the end of a word (as distinct from ‘tion’) to signal it is a derivation of a root morpheme that makes it refer to a person (e.g. music → musician, electric → electrician) (Nunes & Bryant, 2009).

In summary, spelling demands explicit knowledge of the orthographic structure of words, and when compared with reading, spelling development is a protracted process. The foundations of reading and spelling are the same and hence children with reading (decoding) difficulties also have spelling difficulties. However, proficient spelling in most alphabetic languages depends on conditional rules that are only gradually acquired (Pacton & Deacon, 2008; Pacton, Fayol, & Perruchet, 2005), and the letter sequences that are the crux of spelling may be reinforced through writing movements that bring kinaesthetic codes to bear. Against this backdrop, there are several reasons as to why a person might find spelling difficult; however, there are few causal theories that fully address the complexities of the process.

## The relationship between reading and language disorders

Our review so far makes clear that reading (and spelling) disorders are strongly associated with underlying delays and difficulties with language development, a view consistent with earlier epidemiological studies, (e.g. Rutter & Yule, 1975) although these did not differentiate problems of word decoding from reading comprehension. Pine et al. (in press) remind us that in any classification of disorders, it is important to question the extent to which apparently different disorders might reflect the same underlying construct at different points in development. This issue is paramount in relation to the relationship between Learning and Communication Disorders. It is many years since Liberman proposed that ‘reading is parasitic upon language’ (cited by Mattingly, 1972; *p145*); this quotation captures the fact that oral language provides the foundation for written language. Thus, children with difficulties of language and communication are at high-risk of literacy problems (Bishop & Snowling, 2004; Catts & Kamhi, 2005). On the other side of the coin, there is emerging evidence that good language can provide a compensatory resource for children with word-level reading difficulties (Snowling, 2008; Snowling, Gallagher, & Frith, 2003). Moreover, there are likely to be reciprocal interactions such that Learning Disorders will in turn affect the development and outcome of Communication Disorders.

### Continuities between speech, language and reading disorders

It is widely acknowledged that language disorders are heterogeneous, and there is a debate as to how best to classify them (Bishop & Norbury, 2008). DSM-5 distinguishes between Language Impairment (LI), Specific Language Impairment (SLI), Speech-Sound Disorder (SSD) and Social Communication Disorder (associated with pragmatic language difficulties). However, the mapping between these categories and reading disorders is complex. What is clear is that there is considerable overlap, and not only has the co-morbidity with LI been overlooked in many studies of children with dyslexia (McArthur, Hogben, Edwards, Heath, & Mengler, 2000) but also educators have been unaware of the high prevalence of SLI in children with reading comprehension impairments. Given the precedence of speech and language development over the development of written language skills, a strong hypothesis is that the relationship between Communication Disorders and Reading Disorders is one of homotypic co-morbidity (Caron & Rutter, 1991); that is, a Reading Disorder is simply a later manifestation of what was observed earlier as a disorder of spoken language development.

**Literacy skills in speech-sound disorders.** Given evidence of phonological deficits in dyslexia, it is natural to predict that children with speech difficulties affecting the phonological system should be at high-risk of word-level reading difficulties. Contrary to this, speech difficulties actually carry a lower risk of subsequent reading difficulties than do broader language difficulties (see Pennington & Bishop, 2009 for a review). There are a number of possible reasons for this seemingly surprising conclusion. First, speech-sound disorders are typically identified in the preschool years, and for most children, these resolve by the time reading instruction begins. Perhaps reading impairments are seen in children with persistent SSD (Bird, Bishop, & Freeman, 1995; Stackhouse & Snowling, 1992) but not in those whose speech difficulties have resolved (Bishop & Adams, 1990)? Second, the diagnosis of SSD is made on the basis of a child’s speech articulation and their intelligibility. There are likely to be a number of different causes of such difficulties with speech production (including motor impairments), and it is plausible that some but not all of these will compromise learning to read; in particular, it has been proposed that children who make speech errors that are not observed in typical development are at especially high-risk of reading difficulties (Dodd, 1995; Leitão, Hogben, & Fletcher, 1997).

An alternative way of viewing the discrepant findings regarding the co-morbidity of SSD and dyslexia is in terms of shared genetic risk factors (Smith, Pennington, Boada, & Shriberg, 2005). Within this view, children with SSD bring a liability to the task of reading (a heritable endophenotype, Skuse, 2001), which predisposes them to failure, but only when other risk factors are present. In line with this, Peterson, Pennington, Shriberg, and Boada (2009) examined the developmental outcomes of children with SSD at 7–9 years-of-age. The relative risk of literacy disorder in this group was 2.5 times as high as in the general population. Although there was a continuing effect of the persistence of speech problems on phonological awareness (an endophenotype of dyslexia), SSD persistence did not discriminate children who developed literacy difficulties from those who did not. In similar vein, Nathan, Stackhouse, Goulandris, and Snowling (2004) found an association between persistent SSD and spelling, but not reading problems.

In summary, speech difficulties in the preschool years carry a heightened risk of literacy impairments, with a suggestion that phonological awareness and spelling processes may be more vulnerable to the impact of poor speech than reading skills. Some types of speech difficulty (e.g. those characterized by inconsistency of speech errors) that persist to the time of school entry may lead children to experience problems learning to read, but further research is needed to clarify the nature of the speech-sound disorder in such cases.

**Literacy skills in children with language impairment.** In contrast to SSD, there is ample evidence that language impairment in preschool places a child at high-risk of reading difficulties, particularly if they have low general cognitive ability (Catts, Fey, Tomblin, & Zhang, 2002). In line with this, Peterson et al. (2009) reported that the risk of reading disorder among children with co-morbid speech and language impairment was 7.4 times higher than in the general population. Notwithstanding this, literacy outcomes may be age-dependent (Puranik, Petscher, Al Otaiba, Catts, & Lonigan, 2008) and Bishop and colleagues (Bishop & Adams, 1990; Snowling, Bishop, & Stothard, 2000) reported a higher prevalence of dyslexia at school leaving age than in the primary school years. Moreover, nonverbal IQ was a protective factor, such that those with scores above 100 had better outcome than those with lower general cognitive ability. Contrary to this, Catts, Bridges, Little, and Tomblin (2008) examined the growth of word recognition and reading comprehension in a larger sample of children with LI between 2nd and 10th grade. Similar to previous studies, there was a curvilinear pattern of growth, with more gain in reading occurring between 2nd and 4th grade then between 4th and 10th grade – essentially a plateau in reading growth around 6th grade. Although the LI group differed from typically developing children in their baseline reading scores in second grade, gains in reading over time were essentially parallel with no sign of either ‘catch-up’ or a cumulative reading deficit in the language impaired group.

Aside from problems of reading and spelling, problems of reading comprehension are common among children with language impairments (Botting, Simkin, & Conti-Ramsden, 2006; Catts et al., 2006). Moreover, for some children, the problems of reading comprehension are selective and they resemble ‘poor comprehenders’. Catts et al. (2006) conducted an analysis of the language profiles of poor comprehenders identified in 8th grade, reporting data from kindergarten, 2nd and 4th grade. At each time point, the poor comprehenders showed weak language scores suggesting they experienced a stable language deficit, and one that might plausibly be a cause of their problems in understanding what they read (see Nation, Cooksey, Taylor, & Bishop, 2010, for similar findings).

Together, these findings highlight shared risk factors between LI and both dyslexia and reading comprehension impairment. It is clear that children who reach Grade 2 with language delay are likely to suffer longstanding reading difficulties, and so, every effort should be made to identify such children early and to put in place effective interventions.

**Reading skills in children with pragmatic language impairments.** Children with pragmatic language impairments have difficulties with the socially appropriate use of language, although they may have fluent, complex and clearly articulated expressive language. In DSM-5, these children are described as showing ‘Social Communication Disorder’. Relatively little research has examined literacy skills in children with such an impairment. However, given that text comprehension requires the reader to go beyond what is printed, to make inferences and to understand figurative language (Cain, 2010), such children will be at high-risk of reading comprehension difficulties and may struggle, in particular, to understand fictional texts.

One group of children who typically experience pragmatic language difficulties are children with Autism Spectrum Disorders (ASD). Word-level decoding tends to be a relative strength in this group (Saldaña, Carreiras, & Frith, 2009), whereas reading comprehension impairments are three times more common in ASD than in TD populations (Jones et al., 2009; Nation, Clarke, Wright, & Williams, 2006). Indeed, many children with ASD show the ‘poor comprehender’ profile, and in extreme cases, ‘hyperlexia’ has been reported (see Nation, 1999, for a review). The reason for such fractionation of reading skills remains unknown, but it is likely that executive deficits play a role.

### A dimensional perspective on reading disorders

Viewed together, the findings of studies that have attempted to relate children’s speech and language difficulties to their literacy problems highlight the limitations of a categorical approach. There is not a one-to-one mapping between different Communication and Reading Disorders, but it is clear when viewed within a theory of typical reading development that there are shared risk factors. Indeed, Bishop and Snowling (2004) proposed that to understand the inter-relationships between reading and language impairments, it is necessary to adopt a two-dimensional model of the skills that underpin fluent reading ability. As we have seen, decoding skills are underpinned by phonological abilities, whereas broader oral language skills (vocabulary, grammar and pragmatic abilities) underlie reading comprehension. The outcome of an individual’s literacy development depends upon the status of the phonological and broader oral language skills that they bring to the task of reading. Essentially this dimensional view moves away from categorical diagnoses and highlights the different risk factors (cognitive endophenotypes) for dyslexia versus reading comprehension impairment. Within this view, although categorical diagnoses may be useful to clarify the nature of a child’s additional educational needs, there is actually what might be better characterized as a ‘spectrum’ of reading disorders. This idea of a spectrum accommodates those who fulfil externally agreed diagnostic criteria as well as those with ‘sub-clinical’ levels of reading disorders.

### Co-morbidities between reading and other neurocognitive disorders

Aside from co-morbid language impairments, there is growing recognition that Reading Disorders show high rates of comorbidity with other disorders that affect learning, and the co-occurrence of more than one co-morbid condition is likely in severe cases. The question of co-morbidity has been considered in relation to the revision of DSM-5 which has acknowledged the usefulness of dimensional approaches across the range of mental disorders (Helzer et al., 2008; Pine et al., in press). Given that epidemiological studies of learning disorders are rare, evidence about the exact rates of different comorbidities is limited. Dyslexia has been reported to be frequently comorbid with ADHD (Willcutt & Pennington, 2000), developmental coordination disorder (Kadesjo & Gillberg, 2001; Kaplan, Wilson, Dewey, & Crawford, 1998) and mathematics disorder (Dyscalculia in DSM-5) (Landerl & Moll, 2010; Lewis, Hitch, & Walker, 1994). Evidence about co-morbidities with Reading Comprehension Impairment is largely lacking, although as noted above, reading comprehension impairments are observed in ADHD and common in ASD.

The comorbidity between dyslexia and ADHD is perhaps the best researched of the comorbidities. One plausible hypothesis is that their frequent co-occurrence reflects a referral bias, but epidemiological data suggest that this is not the case (Carroll, Maughan, Goodman, & Meltzer, 2005). In addition, there is good evidence that reading (decoding) problems and ADHD reflect shared genetic risk factors that influence the development of both disorders (Willcutt et al., 2003). ADHD is defined in terms of problems on two partially separate dimensions (inattention and hyperactivity/impulsivity), and the shared genetic risk factors are thought to operate more strongly to co-determine symptoms of inattention and reading than hyperactivity and reading (Willcutt et al., 2003).

To understand how co-morbid ADHD may affect learning to read it is important to consider the cognitive impairments associated with the condition. At the cognitive level of explanation, evidence concerning the causes of co-morbidity is mixed. Castellanos and Tannock (2002) proposed that a candidate ‘endophenotype’ underlying both reading disorder and ADHD was poor time perception. However, Gooch, Snowling, and Hulme (2011) compared children with attention or reading difficulties with a co-morbid group and found that problems of time reproduction were associated exclusively with ADHD, whereas problems of phonological awareness were associated exclusively with dyslexia. Children who experienced co-morbid ADHD and dyslexia were in each case the most impaired, but the disorders were additive in their effect.

The relationship between dyslexia and ADHD has also been investigated by McGrath et al. (2011) in a large sample of twins selected for having ADHD and/or reading disorders. In this sample, phonological awareness was specifically related to variations in word reading ability, whereas ‘inhibition’ was a predictor of symptoms of both inattention and hyperactivity/impulsivity. Perhaps most interestingly, a processing speed factor was a predictor of both reading ability and rated inattention, and this shared predictive relationship accounted for their phenotypic correlation. This study therefore provides evidence that symptoms of ADHD and decoding problems may both be influenced by a common cognitive deficit in processing speed. In other words, speed of processing is a putative risk factor for dyslexia, but at present, longitudinal data are lacking.

The nature of the co-morbidity between dyslexia and developmental coordination disorder (DCD) is less clear. On the face of it, there is no reason to predict that children who have difficulties with gross or fine motor skills should have specific difficulties learning to read, although clinical experience suggests that problems of spelling and writing are more likely to occur. On the other hand, the co-morbidity of dyslexia and DCD may turn on deficits shared with other disorders including LI, (in which coordination problems are extremely common, Hill 2001), and ADHD (Raberger & Wimmer, 2003). Indeed, a meta-analysis by Rochelle and Talcott (2006) showed balance deficits in dyslexia were more closely associated with ADHD than with reading problems. Thus, it seems clear that there is no direct causal relationship between impairments of motor development and dyslexia; motor problems may nonetheless be useful as a nonspecific behavioural marker of learning disorder.

### Evidence for multiple risk factors in reading disorders

The idea that there is more than one cognitive ‘endophenotype’ associated with dyslexia suggests that the phonological deficit view that has dominated the field for many years is inadequate. More generally, single deficit accounts of disorders have fallen from favour, and are being replaced by theories that posit multiple deficits underpinning categorical disorders (Bishop, 2006; Pennington, 2006). An important characteristic of cognitive endophenotypes is that they are heritable, and that they are observed in both affected and unaffected family members (Bearden & Freimer, 2006; Skuse, 2001). As they are normally distributed in the population, they can be considered markers of risk, conforming to the idea that disorders are dimensional. A growing body of literature following the development of children who are ‘at risk’ of dyslexia by virtue of having one affected parent, speaks directly to this issue and underlines the view that there is a spectrum of reading disorders.

Family studies of dyslexia are important because, by investigating differences between children from dyslexic and nondyslexic families before they enter formal schooling, they can elucidate the developmental precursors of learning disorders. Furthermore, the comparison of affected and unaffected family members allows the identification of probablistic risk factors that may predispose a child to the disorder (but not lead to it when additional risks are absent). Within this view, children with single deficits are less likely to succumb to failure than children with multiple risk factors.

Contrary to the strict version of the phonological deficit hypothesis, family risk studies have shown that in the preschool years, children who go on to be classified as dyslexic experience a wide range of oral language difficulties, affecting vocabulary and grammar as well as phonology (Gallagher, Frith, & Snowling, 2000; Lyytinen et al., 2006; Scarborough, 1990). Furthermore, while all family risk studies report an elevated rate of poor reading and spelling in children at high-risk of dyslexia, an emerging finding is of similarities between the ‘affected’ and ‘unaffected’ high-risk children on a variety of measures including phoneme awareness (Boets et al., 2010; Pennington & Lefly, 2001), letter knowledge and basic spelling skills (Snowling et al., 2003).

Together, these finding indicate that the family risk of dyslexia is continuous rather than discrete, and there is no clear cut-off between ‘dyslexia’ and unimpaired reading. Children vary in their rates of literacy development, and some children who show slow development in the early stages of reading may proceed to become normal readers. In other words, while the phonological deficits that presage literacy problems can be observed among children at family risk of dyslexia, whether they develop dyslexia or not depends on their wider language skills. Thus, children who show poor phonology (one risk factor) in the context of delayed language (a second risk factor), are more likely to develop a reading disorder than those who have poor phonology in the context of normal language development (Snowling, 2011). From a clinical perspective, children with two risk factors are more likely to reach the diagnostic threshold for dyslexia, although others may experience something akin to a ‘sub-clinical’ form of the disorder. If this characterization is correct, then the longer term literacy outcome of children in the family risk group with normal reading at 8 years might be poor relative to peers – that is, the tendency to ‘compensate’ early may lead to what has been called ‘illusory recovery’ (Scarborough & Dobrich, 1990) and difficulties may re-emerge when the demands of literacy increase.

There is some limited evidence in support of this hypothesis. Snowling, Muter, and Carroll (2007) followed up children at family risk of dyslexia in early adolescence, aged 12–13 years. The children who were defined as ‘dyslexic’ at age 8 years continued to have significant difficulties on all literacy measures. Importantly, however, children who had shown poor performance on tests of phonological skills at an earlier age, but who had not been classified as ‘dyslexic’ now showed deficits in relation to controls in reading fluency, exception word reading and spelling. These children from family risk samples conform to what might be called the ‘broader phenotype of dyslexia’ (or compensated dyslexia; Ramus et al., 2003) – a partial version of the dyslexia phenotype which, in childhood, does not reach diagnostic threshold.

Snowling (2008) tested this ‘multiple deficit’ view further using data from this family study by examining performance on tests tapping visuo-spatial skills, attention control and oral language abilities. As was predicted, individuals with a greater number of cognitive deficits had the worst literacy outcome, and it was more common for the dyslexic group to have multiple impairments than the group who showed the ‘broader phenotype’. Interestingly in the light of probable shared risk factors for learning disorders, although children with the broader phenotype were free of language impairments, it was common for them to experience problems of attention control, and many were considered by their parents to be under-achieving at school.

There is relatively little information about the reading comprehension skills of children and young people from families at high-risk of dyslexia. According to the two-dimensional view, it is unlikely that problems of reading comprehension will reach a ‘diagnostic’ threshold in dyslexia unless broader language skills are also impaired. However, it is perhaps important to note that some children at family risk with impairments of attention would be at more risk of difficulties in text comprehension (McInnes et al., 2003).

In summary, a proper understanding of the co-morbidity between reading disorders and other neurocognitive disorders depends upon identifying endophenotypes for each disorder. The search for such endophenotypes is at a relatively early stage, but where progress has been made, it is becoming clear that the effects of cognitive endophenotypes accumulate to increase the probability of reading impairment.

## The classification of reading and related disorders

The evidence we have reviewed supports a number of important conclusions. Most simply, there are at least two different forms of reading disorder in children: dyslexia (decoding problems) and reading comprehension impairment. Although these disorders are usually defined categorically, it is important to emphasize that reading skills show a continuous distribution in the population and each of these disorders is probably better thought of in dimensional terms – children with dyslexia and reading comprehension impairment are simply at the low end of the distribution of decoding and reading comprehension skills respectively. We have also emphasized that both these different forms of reading difficulty can be seen as reflecting problems with language development, and reading disorders are highly comorbid with diagnoses of language impairment. In addition, reading disorders are frequently comorbid with a range of other seemingly disparate disorders including disorders of attention and motor development. Clinicians therefore should take steps to assess a wide range of potential difficulties in children referred for reading problems, but they should also be clear that many such comorbidities may not be causally related to the reading disorder (this does not deny the potential importance of identifying the other difficulties a child may have and providing appropriate treatment for such difficulties).

It is appropriate to relate these conclusions to the diagnostic categories proposed in DSM-5. Within Neurodevelopmental Disorders, DSM-5 separates Learning Disorders (including Dyslexia and Disorders of Written Expression) from Communication Disorders (including Speech-Sound Disorder, Language Impairment (including Late Language Emergence, Specific Language Impairment, Social Communication Disorder and others).

In the current draft, it is notable that there is no listing for either Reading Comprehension Impairment or for Spelling Disorder. We discuss each of these points in turn before considering the issue of comorbidity. First, it is plain from our review that we disagree with the recommendation:

‘that reading comprehension per se be omitted from DSM-5, because individuals who have specific reading comprehension problems in the presence of good decoding skills, do not meet criteria for dyslexia. Such individuals typically are found to have poor oral language (as in communication disorders). However, specific reading comprehension disorders could be coded under the newly proposed superordinate category of learning disability’.

This recommendation seems to us confusing, because in terms of its diagnosis and treatment, reading comprehension impairment needs to be considered as a related but contrasting disorder to dyslexia.

Likewise, DSM-5 does not propose a separate classification for Spelling Disorder. This decision may reflect the fact that children seldom present for clinical assessment with spelling difficulties in the absence of reading problems. However, there are exceptions (Frith, 1980; Goulandris & Snowling, 1991), and particularly in regular orthographies, a double dissociation can be seen between reading and spelling impairments (Moll & Landerl, 2009). The only reference to spelling difficulty in DSM-5 is as a feature of language impairment where it is ‘banded’ with difficulties in written formulation:

LI affects acquisition and use of spoken language (sound-, word-, sentence, and discourse-level comprehension, production and awareness), written language (reading decoding and comprehension; spelling and written formulation), and other modalities of language (e.g. sign language).

The entry for ‘Disorder of Written Expression’ may well be more specific about spelling difficulty, but at the time of writing, the criteria are still under development. Moreover, there is clearly a danger that the confusion between reading accuracy and reading comprehension, which has been removed in DSM-5 will re-emerge as a confusion between disorders of spelling at the word-level (the ability to recreate the legal sequences of printed words) and of written expression, which draws on a wider range of language skills and control processes. We believe that there is enough evidence to show that disorders of spelling can be observed in isolation from reading disorders and given this, a separate category is warranted.

We also believe that there are important reasons for bringing together disorders of reading and language in a classification system. In addition, given issues concerning the stability of diagnostic criteria that rely upon measuring reading attainments using behavioural tests, there is good reason to include, for each disorder, a cognitive description of the phenotype which will remain the same over development despite possible changes in behavioural manifestation (Morton & Frith, 1995). As the model proposed by Bishop and Snowling (2004) suggests, it is productive to consider a dimensional approach to the classification of reading impairments such that oral language difficulties in the phonological domain place a child at risk of decoding deficits (dyslexia) whereas wider oral language difficulties (particularly including semantic and grammatical difficulties) place a child at risk of reading comprehension difficulties. While ideally, a dimensional scheme should incorporate what is known of other risk factors, an important spin-off of the two-dimensional model is that it has direct implications for screening, early identification and intervention. The broad aim of this article has been to situate reading disorders within the context of language difficulties; it will fall to clinicians to make these links if they are not explicitly acknowledged in the classification system.

### Clinical and educational implications

We have outlined the case that there are two quite distinct forms of reading difficulty in children (dyslexia and reading comprehension impairment), both of which are quite common; spelling difficulty is a key feature of dyslexia, but such difficulties can also occur in isolation. Dyslexia appears to be caused primarily by an underlying weakness in phonological (speech sound) processing whereas reading comprehension impairment appears to reflect broader language processing weaknesses affecting a wide range of skills including vocabulary, grammar, listening comprehension and narrative skills. The difficulties experienced by children with reading comprehension impairment appear to be on a continuum with children who would qualify for a diagnosis of language impairment. However, we have also argued that considering these two disorders in categorical terms is a limited view; the skills which underlie decoding, spelling and reading comprehension are continuously distributed in the population. The literacy outcome for an individual depends upon the interaction of their cognitive strengths and difficulties, primarily with respect to these dimensions, although it is likely that additional deficits (e.g. in executive skills or in speed of processing) will also play a role.

**Assessment of reading disorders.** For many years, dyslexia was considered to be a ‘specific’ learning difficulty which affected reading (and spelling), but not other general cognitive abilities. Accordingly, it was defined using a ‘discrepancy’ approach: that is, measured reading attainment should be below the level expected based on age and IQ (Rutter & Yule, 1975). However, building on a variety of evidence, the role of IQ in explaining reading (decoding) problems has been rejected (e.g. Hatcher & Hulme, 1999; Shaywitz, Fletcher, Holahan, & Shaywitz, 1992; Stanovich & Siegel, 1994). In line with this, DSM-5 explicitly states that reading attainment need not be out of line with general cognitive ability (although it is noteworthy that ‘the term dyslexia *might be used* where there is evidence of a discrepancy’– this caveat is important to accommodate cases of dyslexia in high-functioning individuals in whom, reading deficits are mild or compensated as we have argued in relation to the ‘broader phenotype’).

It follows that DSM-5 recommends reading should be assessed as follows:

Multiple sources of information are to be used to assess reading, one of which must be an individually administered, culturally appropriate and psychometrically sound standardized measure of reading and reading-related abilities.

Our review highlights the need to ensure that tests of reading fluency and comprehension are included to ensure the nature of the disorder is clarified – we note here that problems of reading fluency are far more common in readers of the European alphabetic languages than are problems of reading accuracy, reflecting the relative ease of learning to decode in these orthographies. Furthermore, the recommendation does not detail ‘reading-related abilities’; we would argue that information critical to intervention should be gathered from tests of phonological awareness and related skills, such as rapid naming (particularly important for dyslexia) and oral language skills (particularly important where there are problems of reading comprehension impairment) (Muter & Snowling, 2010). A single word spelling test, separate from written expression, is also important to differentiate dyslexic difficulties from problems of language expression and or motoric skills.

We would note that ‘diagnosis’ inevitably involves placing arbitrary divisions on what is essentially a continuous distribution of reading skills in the population. Although, administratively it may sometimes be useful and convenient to categorize children as being ‘dyslexic’ or a ‘poor comprehender’, in reality, there are in the population continuous variations in reading and other cognitive skills underlying a ‘spectrum’ of reading difficulties. This has implications for the validity of diagnostic labels (Rutter, 2011). The longitudinal stability of a ‘dyslexia’ diagnosis is low (Shaywitz et al., 1992) and this is true at all levels of severity of the disorder (Wagner, Brown Waesche, Schatschneider, Maner, & Ahmed, 2011). Moreover, there is little agreement between traditional behavioural definitions of dyslexia and definitions which use ‘response to intervention’ as a metric (Brown Waesche, Schatschneider, Maner, Ahmed, & Wagner, 2011). Such findings caution against the use of a diagnostic category to describe an individual whose learning disorder is prone to change with age and in response to intervention (Fletcher et al., 2007).

Finally, assessing reading and related skills only touches the surface of some of the complex issues surrounding the diagnosis of reading disorders. Following a recent review for the UK government, Rose (2009) stated that ‘Dyslexia....is best thought of as a continuum, not a distinct category, and there are no clear cut-off points. Co-occurring difficulties may be seen in aspects of language, motor co-ordination, mental calculation, concentration and personal organization, but these are not, by themselves, markers of dyslexia.’ Thus, while there is good agreement that dyslexia significantly impedes learning, continuities and co-morbidities with other language and learning disorders complicates identification, assessment and diagnosis. A similar argument could be made for reading comprehension impairment.

**Interventions for children’s reading difficulties.** The different causes of dyslexia and reading comprehension impairment clearly imply that different forms of intervention will be required to help children with these different forms of reading disorder. The different forms of intervention required to reinforce the importance of the correct assessment and diagnosis of children’s reading difficulties.

There is now a considerable body of evidence about effective interventions for children with dyslexia, and a gradually increasing body of evidence concerning interventions for reading comprehension impairment (for recent reviews see Duff & Clarke, 2011; Fletcher et al., 2007; Snowling & Hulme, 2011). As might be expected from the theoretical framework we have discussed, children with dyslexic difficulties benefit from teaching that directly targets learning spelling-sound relationships and helps to overcome their phonological difficulties (Bus & van Ijzendoorn, 1999; Torgesen, 2005), whereas children with reading comprehension impairments require interventions that work on the oral language skills that underlie the condition (Clarke, Snowling, Truelove, & Hulme, 2010; National Reading Panel, 2000).

A natural question is whether, if children could be identified early as being ‘at risk’ of reading difficulties, interventions could prevent the development of dyslexia and/or reading comprehension impairment. The evidence we have so far is limited, but does suggest that later reading problems might at least be reduced by suitable early interventions. There is a long history of studies showing that early phonological training can benefit the development of decoding skills (Bradley & Bryant, 1978; Lundberg, Frost, & Petersen, 1988). One of the first randomized trials to address this issue was conducted by Bowyer-Crane et al. (2008) who evaluated early interventions to ameliorate both decoding and language comprehension difficulties. This study compared a Phonology with Reading programme (P + R) to an Oral language Programme (OL) delivered by specially trained teaching assistants to children who were selected for having weak oral language skills at school entry. The children who received the P + R programme did significantly better on tests of phoneme awareness, letter-sound knowledge, basic reading and spelling skills than children who received the OL programme, whereas those who received the OL programme did significantly better on tests of vocabulary and grammar, and there was a trend for more improvement in narrative skills. Moreover, the relative gains for both groups were maintained some 5 months after the intervention had ceased. A very important question, that is still virtually unaddressed, is the extent to which such interventions can have long-term effects, and there is a pressing need for large scale randomized trials of long duration to evaluate this issue.

## Conclusions

We have outlined evidence that there are two different forms of reading disorder in children (dyslexia and reading comprehension impairment). Both disorders are best thought of in dimensional terms, and both can easily be diagnosed and treated. We have also emphasized that reading disorders typically arise from underlying language weaknesses which may not be severe enough in themselves to merit a clinical diagnosis of a language or speech disorder. Nevertheless, reading, language and speech disorders share many features and in all likelihood arise from common shared risk factors. Our proposal for making more explicit links in DSM-5 between Communication and Learning Disorders emphasises the similarities between these disorders in their behavioural characteristics, aetiologies and the forms of treatment that are required. However, our main plea is for explicit recognition that there is more than one form of reading disorder, and careful assessment is required to ensure appropriate intervention. Whilst it could be argued that there is insufficient evidence that reading comprehension is distinct from Language Impairment, for it to be included as a distinct disorder in DSM-V, there is no doubt that it can be reliably diagnosed and that it causes functional impairment, two of the cardinal requirements underpinning the revision of DSM-4. Similar points have been made by some of the foremost researchers in the field of reading and reading intervention (Fletcher, Lyon, Fuchs, & Barnes, 2011). We strongly agree that a more inclusive classification of ‘Learning Disorders’ is required, and to fail to take account of this will be to the detriment of substantial numbers of children in our educational system.
